# Generation of human induced pluripotent stem cell lines derived from Wolf–Hirschhorn syndrome patients with chromosomal 4p deletion

**DOI:** 10.1007/s13577-025-01292-x

**Published:** 2025-09-19

**Authors:** Tomoya Shimizu, Miho Takami, Mami Matsuo-Takasaki, Michiya Noguchi, Yukio Nakamura, Tadayoshi Hayata, Yohei Hayashi

**Affiliations:** 1 iPS Cell Advanced Characterization and Development Team, BioResource Research Center, RIKEN, 3-1-1 Koyadai, Tsukuba, Ibaraki 305-0074 Japan; 2 Department of Molecular Pharmacology, Graduate School of Pharmaceutical Sciences and Faculty of Pharmaceutical Sciences, Tokyo University of Science, 6-3-1 Niijuku, Katsushika, Tokyo, 125-8585 Japan; 3 Cell Engineering Division, BioResource Research Center, RIKEN, 3-1-1 Koyadai, Tsukuba, Ibaraki 305-0074 Japan; 4 CiRA Foundation, Research and Development Center, Nakanoshima Qross 7F, 4-3-51 Nakanoshima, Kitaku, Osaka, 530-0005 Japan

**Keywords:** Human induced pluripotent stem cells, Wolf–Hirschhorn syndrome (WHS), Self-renewal, Pluripotency, Chromosomal deletion

## Abstract

Wolf–Hirschhorn syndrome (WHS) is a devastating congenital disease caused by deletions on the short arm of chromosome 4 (4p), for which no curative treatments currently exist. To facilitate the development of therapeutic strategies, the development of experimental models of WHS is crucial for investigating its etiology and pathogenesis, which remain elusive. In this study, we successfully generated human induced pluripotent stem cells (hiPSCs) from three fibroblast lines from WHS patients. We then characterized these hiPSCs, along with one hiPSC line previously generated from peripheral blood mononuclear cells, as part of a Japanese nationwide project. All four hiPSC lines exhibited characteristics of self-renewal, pluripotency, and karyotypes with expected 4p deletions. Copy number variation microarray analysis revealed that these WHS-specific hiPSCs carried hemizygous deletions in p15.1–p16.3 regions, commonly encompassing 100 genes. Transcriptome analysis showed that the expression of these genes faithfully reflected hemizygous deletion in these WHS-specific hiPSCs and that these down-regulated genes were associated with the development of neural crest cells. These results indicate that WHS-specific hiPSCs can recapitulate the abnormal genomic structure genes related to and the gene expression profile observed in WHS patients. Given the limited understanding of the molecular pathogenesis of WHS, these cellular resources will be instrumental in modeling disease phenotypes and in advancing novel therapies for this syndrome.

**Supplementary Information:**

The online version contains supplementary material available at 10.1007/s13577-025-01292-x.

## Introduction

Chromosomal deletions are major causes of genetic disorders, often resulting in congenital malformations, intellectual disabilities, and other clinical features. Despite the severity of these diorders, effective or curative therapies remain limited. Pathological models that accurately reflect patients' symptoms are essential for understanding disease mechanisms and developing new therapies. While diseases caused by single-gene mutations are widely modelled using genetically modified mice, diseases caused by chromosomal deletions are challenging to be modelled using experimental animals due to the difficulty of precisely reproducing large-scale gene deletions and to differences in chromosomal structure across species. Therefore, pathogenic mechanisms caused by chromosome deletions largely remain elusive to be investigated in the biomedical field.

Disease-specific human induced pluripotent stem cells (hiPSCs), generated from somatic cells of patients with genetic disorders, offer valuable tools for studying disease mechanisms and drug discovery. These hiPSCs can be differentiated into various tissues, allowing us to model disease phenotypes while maintaining patient-specific genetic mutations. Since this approach is particularly effective to chromosomal abnormalities, patient-derived hiPSCs possess the same chromosome abnormalities as the patient, enabling the development of disease models that mimics the patient's abnormal phenotypes [1]. Our group has previously generated hiPSCs from patients carrying ring chromosomes [2], juvenile nephronophthisis with 2q13 deletion [3], and DiGeorge syndrome with 22q11.2 deletion [4].

In this study, we focus on Wolf–Hirschhorn syndrome (WHS; 4p deletion syndrome; OMIM #194190). This syndrome is caused by a heterozygous deletion of a specific region on the short arm of chromosome 4 (4p). Within this deleted region, several candidate genes, including *NSD2* (*WHSC1*) [5, 6], *NELFA* (*WHSC2*) [7], *LETM1* [8, 9], *FGFR3* [10], and *FGFRL1* [11], have been implicated in the pathogenesis of WHS [12]; however, critical genes responsible for the major features of WHS remain to be definitively identified. While a variety of clinical presentations have been reported in patients with WHS, common features of WHS include a characteristic facial appearance and cleft palate. These craniofacial symptoms may arise from abnormalities in neural crest cell (NCC) development, a transient process during embryogenesis [12, 13]. Studies using amphibian embryos have shown that the disruption of WHS-related genes leads to abnormal NCC function and altered facial morphologies [14, 15]. However, these models have limitations as they typically examine the loss of function of a single gene, whereas actual WHS patients have multiple genes deleted simultaneously. While murine models with deletions of genomic regions corresponding to the WHS critical region have been reported [16], the underlying pathogenic mechanisms have not been largely elucidated. Therefore, establishing a model of NCC pathogenesis using cells derived from WHS patients is particularly valuable. Since NCCs are a transient cell population during fetal development, obtaining them directly from WHS patients is challenging. Cell resources that can recapitulate NCC development and function of WHS patients are a prerequisite. In this regard, human induced pluripotent stem cells (hiPSCs) are ideal cell resources as they can differentiate into NCCs and their derivatives.

Here, we report the establishment of hiPSCs derived from WHS patients to investigate their pathogenic mechanisms. These WHS-specific hiPSCs carry 4p deletions and show the decreased expression of many genes within the deleted region while maintaining their self-renewal and pluripotency. These WHS-specific hiPSCs should contribute to the development of improved pathological models and a better understanding of the molecular pathogenesis of WHS.

## Materials and methods

### Human fibroblasts

Fibroblasts from WHS patients, GM00072, GM00343, and GM04126 Lot#B, were purchased from the NIGMS Human Genetic Cell Repository at the Coriell Institute for Medical Research. Control TIG1 fibroblasts (RCB4467) was obtained from RIKEN BioResource Research Center (BRC). Fibroblasts for hiPSC generation were cultured in a fibroblast medium consisted of high glucose Dulbecco's Modified Eagle's Medium (DMEM; Nacalai tesque, Kyoto, Japan) supplemented with 10% FBS (Biosera, Nuaille, France), 1% Pyruvate and 1% penicillin/streptomycin (P/S) solution (Nacalai tesque) at 37ºC and 5% CO_2_. Fibroblasts were cryopreserved with CELLBANKER 1 plus (Zenogen pharma, Fukushima, Japan).

### Generation of iPSCs from fibroblasts with episomal plasmid vectors

hiPSCs were generated from human fibroblasts obtained from WHS patients using episomal plasmid vectors to express *OCT4, SOX2, KLF4, L-MYC, LIN28, mp53DD, and EBNA1* [17, 18]. Fibroblasts were dissociated into single cells with TrypLE Express (Thermo Fisher Scientific, Waltham, MA). After centrifugation at 200 × g for 3 min, 6 × 10^5^ cells were resuspended in 100 µL Buffer R containing 1 µg each of episomal plasmid vectors, pCXLE-hOCT3/4-shp53-F (Addgene #27077), pCXLE-hSK (Addgene #27078), and pCXLE-hUL (Addgene #27080). These cells were electroporated using a Neon transfection system (Thermo Fisher Scientific) with a 100 µL kit, following the manufacturer's instructions (1650 V, width: 10 ms, pulse number: 3). After electroporation, these cells were seeded onto 6-well plates coated with 0.1% gelatin solution (FUJIFILM Wako Pure Chemical Corporation, Osaka, Japan) and cultured for 1 week in the fibroblast medium. Seven days after transfection, the transfected cells were collected with TrypLE Express and re-plated at 1 × 10^5^ cells/well on gelatin-coated dishes co-cultured with mitomycin C (Merck KGaA, Darmstadt, Germany)-treated SNL feeder cells (CBA-316, Cell Biolabs). On the next day, the medium was replaced with Primate ES cell medium (REPROCELL, Kanagawa, Japan) containing 20 ng/ml bFGF (Nacalai USA, San Diego, CA) for on-feeder dishes. Reprogrammed cell colonies with hESC-like morphology were observed and selected for further culture and evaluation approximately 25 days after plating.

### Establishment of iPSC lines and culture

hiPSC-like colonies were picked manually by micropipettes under a microscope and seeded to another feeder-free plate in StemFit AK02N medium supplemented with 0.25 µg/cm^2^ iMatrix (iMatrix-511 silk; Matrixome, Osaka, Japan) and 10 µM Y-27632 (FUJIFILM Wako Pure Chemical Corporation), and cultured at 37 °C and 5% CO_2_. Y-27632 was removed the following day, and the medium was changed every other day until the cells were passaged. The hiPSCs were single-cell passaged every 6–8 days using 0.5 × TrypLE Select (TrypLE Select (Thermo Fisher, Waltham, MA) diluted 1:1 with 0.5 mM EDTA solution) or just 0.5 mM EDTA solution (Nacalai Tesque). Then, cells were seeded at about 2,500 cells/cm^2^ in StemFit AK02N medium supplemented with 10 μM Y-27632 and 0.25 μg/cm^2^ iMatrix [19]. The medium was changed every other day with StemFit AK02N medium starting the next day after passage. For cell counting, the cell solution was diluted with 0.4% trypan blue solution (Merck) to distinguish between live and dead cells and counted using a Cell Counter (OLYMPUS, Tokyo, Japan).

HiPSC clones, HPS3001, 3002, 3003, 3004 and 3005, derived from PBMC of a WHS patient (CiRA-j-1560) were obtained from RIKEN BRC cell bank. These iPSCs were established at the Center for iPS Cell Research and Application (CiRA), Kyoto University and included in CiCLeD (CiRA iPS Cell Line Database; http://cicled.cira.kyoto-u.ac.jp/) for the establishment information [20]. Control hiPSCs derived from 4 healthy donors, HiPS-WTc11(GM25256 in Coriell Institute; a gift from Dr. Bruce Conklin in J David Gladstone Institutes) [21, 22], 19-9-7T [23] obtained from WiCell, 648A1 [18] and 1383D6 [19] generated in CiRA in Kyoto University and obtained from RIKEN BRC, were used in this study.

### Detection of the episomal copy number in these established iPSCs

Genomic DNA (gDNA) was extracted from 5 × 10^5^ to 1 × 10^6^ cells using DNeasy Blood & Tissue Kit (Qiagen, Venlo, NL) according to the manufacturer's instructions. gDNA samples were analyzed with quantitative PCR analysis. A standard curve was generated using the pCXLE-hOCT3/4-shp53-F plasmid to determine the copy number and threshold cycle (Ct) values for the EBNA-1 gene carried in the episomal vectors. Copy numbers of EBNA-1 in each hiPSC sample was calculated from the observed Ct values. Cell numbers per reaction were calculated based on the gDNA concentration and the number of cells collected for gDNA extraction. Quantitative qPCR was performed using a QuantStudio 3 System (Thermo Fisher Scientific) and PowerUp SYBR Green Master Mix (Thermo Fisher Scientific) according to the manufacturer’s instructions. The primer sequences of EBNA-1 are in the Supplementary Table 1.

### In vitro three-germ-layer differentiation assay by forming embryoid bodies (EBs)

EB formation assay was performed following our previous protocol with minor modifications [2–4, 22, 24, 25]. Briefly, 1.2 × 10^6^ cells were suspended in StemFit AK02N (supplemented with 12 μL of 10 mM Y-27632 solution). hiPSCs were collected with a cell scraper after dissociation using 0.5 mM EDTA in PBS. Then, the cells were transferred to V-bottom 96-well plates (10,000 cells/well) in 100 µL of StemFit AK02N medium supplemented with 10 µM Y-27632 and centrifuged at 200 × g for 3 min to form aggregates. On day 2, 100 µL of high-glucose DMEM containing 10% FBS, 1% Pyruvate, and 1% P/S solution was added. On day 4 and 6, 100 µL of the culture medium was changed. On day 8, EBs were transferred into gelatin-coated 12-well plate (24 EBs / well). The medium was changed every other day. On day 16, EB samples were fixed for immunocytochemistry using antibodies against TUJ1 (neuron (ectoderm) marker), SMA (muscle (mesoderm) marker), and AFP (hepatocyte (endoderm) marker).

### In vivo three-germ-layer differentiation assay by forming teratomas

All animal experiments were approved by the Animal Experimentation Committee at the RIKEN Tsukuba Institute and performed according to the institutional guidelines and the “Guide for the Care and Use of Laboratory Animals” published by the National Institutes of Health. Teratomas were formed following our previous studies [2–4, 22, 24, 25]. Four-week-old male NOD.CB17-Prkdc^scid^/J mice were purchased from Charles River Laboratories Japan (Charles River Laboratories, Kanagawa, Japan). hiPSCs were suspended at 1 ~ 2 × 10^6^ cells/mL in StemFit AK02N complete with 10 μM Y-27632, mixed 1:1 with Matrigel (Corning, Corning, NY), and injected subcutaneously into both groins of 7-week-old mice using a sterile 1 mL syringe with a 26G needle. Seventeen weeks post-injection, tumors were surgically dissected, fixed in 4% paraformaldehyde (PFA). Paraffin-embedded sections were generated from the tumors and stained with hematoxylin and eosin by Genostaff inc., Tokyo, Japan.

### Karyotype analysis

hiPSCs were seeded on a 100-mm dish with 10 mL of StemFit AK02N complete supplemented with 10 µM Y-27632 and 0.25 µg/cm^2^ iMatrix-511. Karyotyping samples were obtained by incubating hiPSCs cultured on day 4 ~ 5 after seeding with 10 µL of colcemid (Nacalai Tesque) for 1.5 ~ 2 h at 37 °C. The se cells were then washed in PBS, dissociated with 0.5 mM EDTA in PBS, and collected into a tube with a centrifugation at 1,500 rpm for 5 min. Cell pellets were suspended in 3 mL of 0.075 M KCl (Nacalai Tesque) solution at room temperature for 15 min as a hypotonic treatment. These cells were then gradually fixed with Carnoy’s solution (methanol: acetic acid = 3:1) (Nacalai Tesque). Six mL of Carnoy’s solution was added gently, and the samples were centrifuged at 1600 rpm for 7 min three times, and finally suspended in 1 mL of Carnoy’s solution. Q-banding analysis was performed by Chromocenter Inc., Tottori, Japan.

### Immunocytochemistry and flow cytometry

Immunocytochemistry and flow cytometry were performed as previously described [2–4, 22, 24, 25] at passage number 5-15. For immunocytochemistry, cells were fixed with 4% PFA and washed with PBS. They were permeabilized with 0.1% Triton X-100 (Wako) in PBS and blocked with 1% bovine serum albumin (BSA;FUJIFILM Wako Pure Chemical Corporation) in PBS. These samples were incubated with primary antibodies at 4 ºC overnight, washed three times with PBS, and then incubated with secondary antibodies at room temperature for 1 h. After three additional PBS washes, the nuclei were stained using Fluoro-KEEPER Antifade Reagent Non-Hardening Type with DAPI (Nacalai Tesque). Images of the cells were captured using a BZ-X800 fluorescence microscope (Keyence). For flow cytometry, the cells were dissociated using Accutase (Nacalai Tesque), resuspended in a custom buffer (0.5 mM EDTA in PBS supplemented with 1% BSA), and incubated for one hour with antibodies. These stained cells were analyzed using an SH800S cell sorter and accompanying software (Sony, Tokyo, Japan). Primary and secondary antibodies used in this study are listed in Supplementary Table 1.

### Copy number variation/comparative genomic hybridization array analysis

Comparative genomic hybridization (CGH) array analysis was used to detect genome-wide DNA copy number variations. Genomic DNA was extracted from WHS-specific iPSCs using the DNeasy Blood & Tissue Kit. CGH array analysis was performed by DNA Chip Research Inc., Knagawa, Japan.

### Quantitative RT-PCR

Total RNA was extracted using Fastgene RNA Premium Kit (Nippon Genetics, Tokyo, Japan) following the manufacturer's instructions. cDNA were synthesized using ReverTra Ace qPCR RT kit (TOYOBO, Osaka, Japan), following the manufacturer’s instructions. Real-time qPCR reactions were performed with a QuantStudio 3 system using THUNDERBIRD Probe qPCR Mix (TOYOBO) with TaqMan probes (Thermo Fisher Scientific), listed in Supplementary Table 1. The relative values of gene expression were calculated as fold changes relative to the control (ΔΔCt method) after normalization to the expression value of GAPDH.

### RNA-seq

Total RNA was extracted from hiPSCs using the FastGene RNA Premium Kit following the manufacturer's instructions. Non-directional libraries were constructed and were sequenced on an Illumina NovaSeq600 (Illumina, Inc, CA, USA), generating 150 bp paired-end reads with an average yield of 20 million reads (6 Gb) per sample. The raw sequencing data were processed and analyzed using CLC Genomics Workbench v20.0.2 (QIAGEN, Hulsterweg, the Netherlands). The analysis workflow included quality control, read mapping to the reference genome (hg19 Homo sapiens), transcript quantification, and differential expression analysis. Gene ontology analysis was performed with Metascape [26]. Visualization of the results, including volcano plot and bar plots, was performed using R software (version 4.4.3) with ggplot2 and dyplyer packages.

### STR analysis

STR analysis was performed following our previous studies [2–4, 22, 24, 25] on hiPSC lines and original fiboblast lines. Genomic DNA were extracted from hiPSCs with DNeasy Blood & Tissue and analyzed using PowerPlex 16 System (Promega, Madison, WI) for STR-PCR.

### Mycoplasma tests

Indirect DNA fluorescent staining and nested PCR were performed on hiPSC samples as previously described [27–29]. The hiPSC culture medium was tested for mycoplasma contamination by staining with bisBenzimide H 33258 (Sigma-Aldrich) after 5–6 days of co-culture with VERO cells (RCB0001, RIKEN BRC Cell Bank), which served as mycoplasma infection indicator cells. DNA samples were extracted and analyzed using nested PCR at approximately passage number 10. For the PCR, AmpliTaq Gold 360 DNA Polymerase (Thermo Fisher Scientific) was used. The same thermocycling conditions were applied for both PCRs: an initial denaturation at 95 °C for 10 min, followed by 30 cycles of thermocycling (30 s at 95 °C, 2 min at 55 °C, and 2 min at 72 °C) with a final extension at 72 °C for 5 min. PCR products were analyzed with electrophoresis using 2% agarose gel stained with ethidium bromide. The primer sequences used for the PCR are shown in Supplementary Table 1.

## Results and discussion

### Generation and characterization of hiPSCs derived from WHS patients

**Fig. 1:**
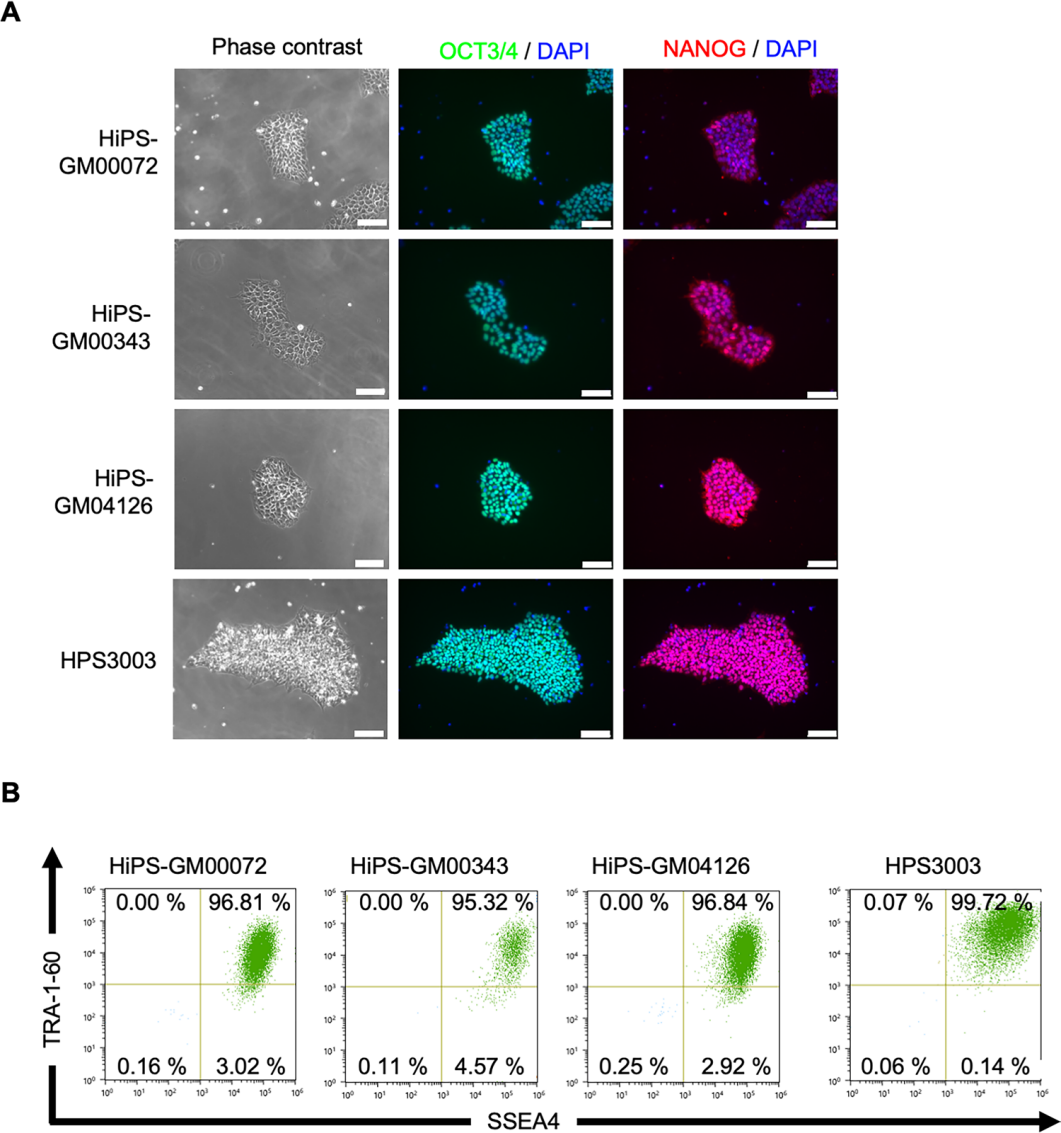
The expression of self-renewal markers in hiPSC lines derived from WHS patients. **A** Phase contrast images and immunocytochemistry of hiPSCs derived from WHS patients. Phase contrast images (left) and immunocytochemistry for self-renewal markers OCT3/4 (middle) and NANOG (right). Scale bars, 100 µm. **B** Flow cytometry analysis of self-renewal markers, SSEA4 (horizontal axis) and TRA-1-60 (vertical axis)

**Fig. 2:**
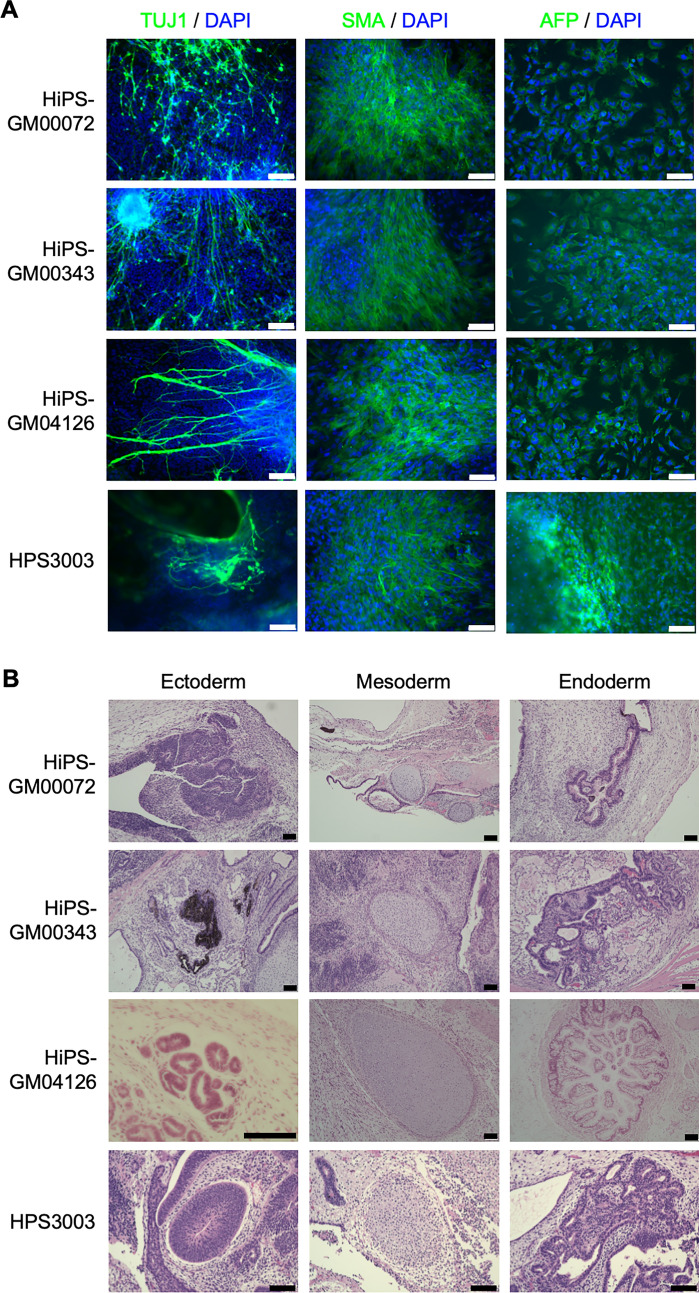
Pluripotency of hiPSC lines derived from WHS patients. **A** Immunocytochemistry of TUJ1, SMA, and AFP on embryoid bodies (EBs) differentiated from WHS-specific hiPSCs. **B** Hematoxylin and eosin staining of teratoma section derived from WHS-specific hiPSCs. Scale bars, 100 µm in **A** and **B**

We obtained three fibroblast lines, GM00072 [30–32], GM00343 [31, 32], and GM04126 [32], derived from WHS patients from the Coriell Institute. The available patient information is summarized in Supplementary Table 2. HiPSCs were generated from these fibroblast lines with the transfection of episomal plasmid vectors carrying reprogramming factors. Approximately 25 days after the transfection of episomal plasmid vectors by electroporation, hiPS-like colonies were observed and picked up. These colonies were expanded and established as clonal hiPSC lines from each patient’s cells. Among these clones, one clone from each of the fibroblast lines was selected for further experiments, for which the absence of the episomal plasmid vectors was confirmed (HiPS-GM00072 #2, HiPS-GM00343 #4, and HiPS-GM04126 #5) (Supplementary Fig. 1A–C). STR-PCR analysis confirmed that these hiPSC lines were made from the same donors as the original fibroblast lines (Data not shown due to individual privacy). For the subsequent characterization, we added a WHS-specific hiPSC line from another patient, HPS3003, established in a previous Japanese nationwide study [20]. This hiPSC line was also confirmed to be free of residual episomal plasmid vectors in the original study. All four WHS-specific hiPSCs were tested negative for mycoplasma contamination (Supplementary Fig. 2). These WHS-specific hiPSCs exhibited typical morphologies of human pluripotent stem cells (Fig. 1A) and expressed self-renewal markers, such as OCT3/4, NANOG (Fig. 1A), SSEA-4, and TRA-1-60 (Fig. 1B). To assess the pluripotency of these hiPSCs, embryoid bodies (EB) were formed to allow for differentiation into three germ layers in vitro. After 16 days of EB formation, the expression of TUJ1 (ectodermal marker), SMA (mesodermal marker), and AFP (endodermal marker) was detected (Fig. 2A). These results indicated that these WHS-specific hiPSCs had the capacity to differentiate into all three germ layers in vitro. Furthermore, hiPSCs were injected into immunodeficient mice to form teratomas. Teratomas derived from WHS-specific hiPSCs contained tissues from all three germ layers, including neuroepithelium (ectoderm), melanocytes (ectoderm), cartilage (mesoderm), and gastrointestinal-like structures (endoderm) (Fig. 2B). These results indicate that these WHS-specific hiPSCs maintain self-renewal ability and pluripotency without residual transgenes.

### Identification of deleted genes in WHS-specific hiPSCs

**Fig. 3:**
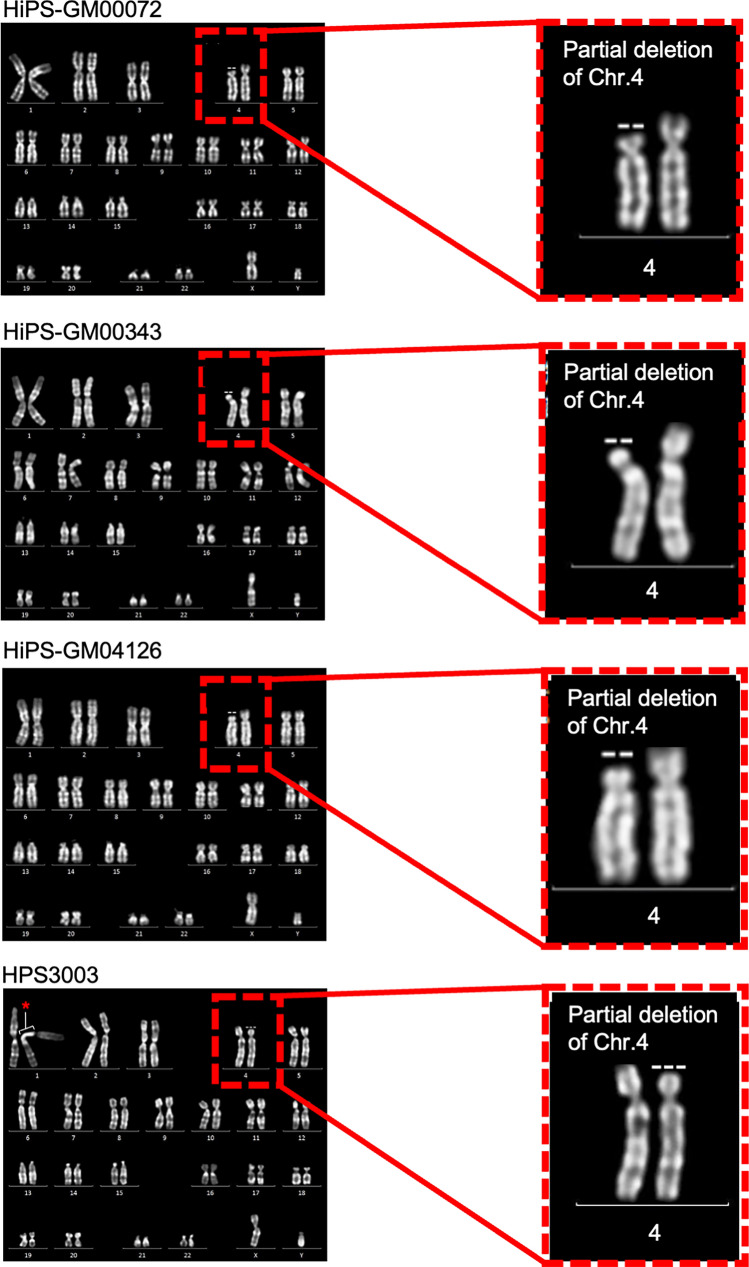
Hemizygous deletions of chromosome 4 in hiPSC lines derived from WHS patients. Representative images of karyotype detected with Q-banding assays for HiPS-GM00072, HiPS-GM00343, HiPS-GM04126, and HPS3003. Enlarged images show a partial deletion in the short arm of chromosome 4. *Indicates the extension of heterochromatin region

**Fig. 4:**
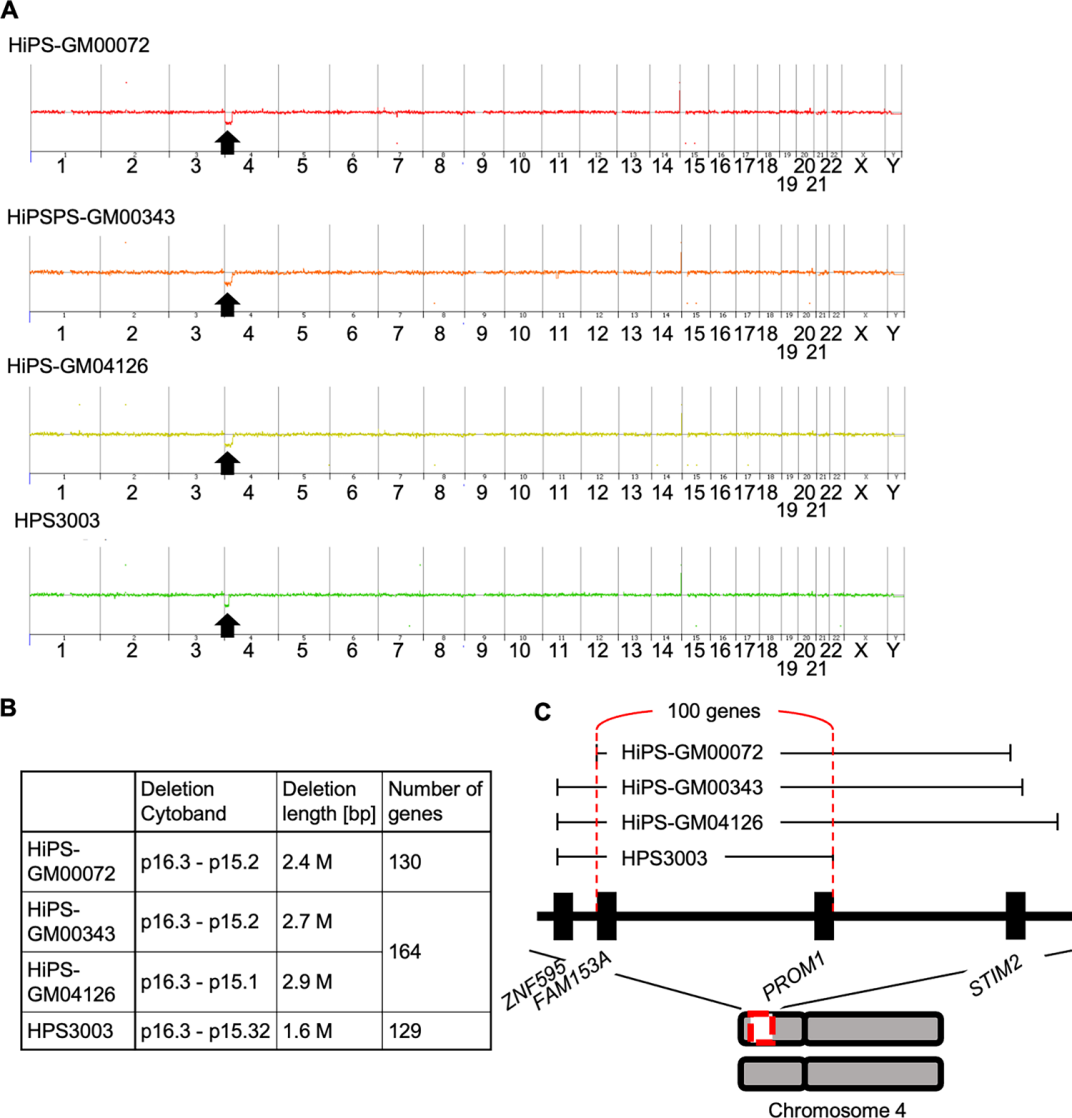
Detection of chromosomal deletions in hiPSC lines derived from WHS patients. **A** Whole genome view of CGH array analysis. Arrowheads indicate decreased copy number in the 4p region. **B** Summary of chromosomal deletions in the 4 WHS-specific hiPSC lines. **C** Schematics show that 100 genes are commonly deleted in the 4 WHS-specific hiPSC lines

To confirm that WHS-specific hiPSCs retain the chromosomal abnormality carried in these patients, karyotyping was performed. Quinacrine-based fluorescent band (Q-band) karyotyping showed a hemizygous deletion of the 4p region with no other chromosomal abnormalities in these WHS-specific hiPSCs (Fig. 3). Specifically, this deletion was observed in 10/10 cells analyzed for HiPS-GM00072, 9/9 cells analyzed for HiPS-GM00343, 9/9 cells analyzed for HiPS-GM00343, and 9/9 cells analyzed for HPS3003. Copy number variation (CNV) analysis was then performed to identify the specific genes deleted within the 4p region (Fig. 4A). As a result, these hiPSCs had hemizygous deletions from p15.1 to p16.3 on chromosome 4 without any noticeable copy number changes elsewhere in the genome. These results were consistent with the results of karyotyping and/or CNV microarray analysis obtained from original fibroblasts in a previous study [32]. The size of the deleted regions varied among these cell lines; HiPS-GM00072 had a 2.4 M bp deletion spanning 130 genes; HiPS-GM00343 and HiPS-GM04126 had 2.7 M bp and 2.9 M bp deletions spanning 164 genes each; HPS3003 had a 1.6 M bp deletion spanning 129 genes (Fig. 4B and Supplementary Table 3). In total, 100 genes in the 4p region were commonly deleted in all 4 WHS-specific hiPSC lines (Fig. 4C).

### The abnormality of gene expression in WHS-specific hiPSCs

**Fig. 5:**
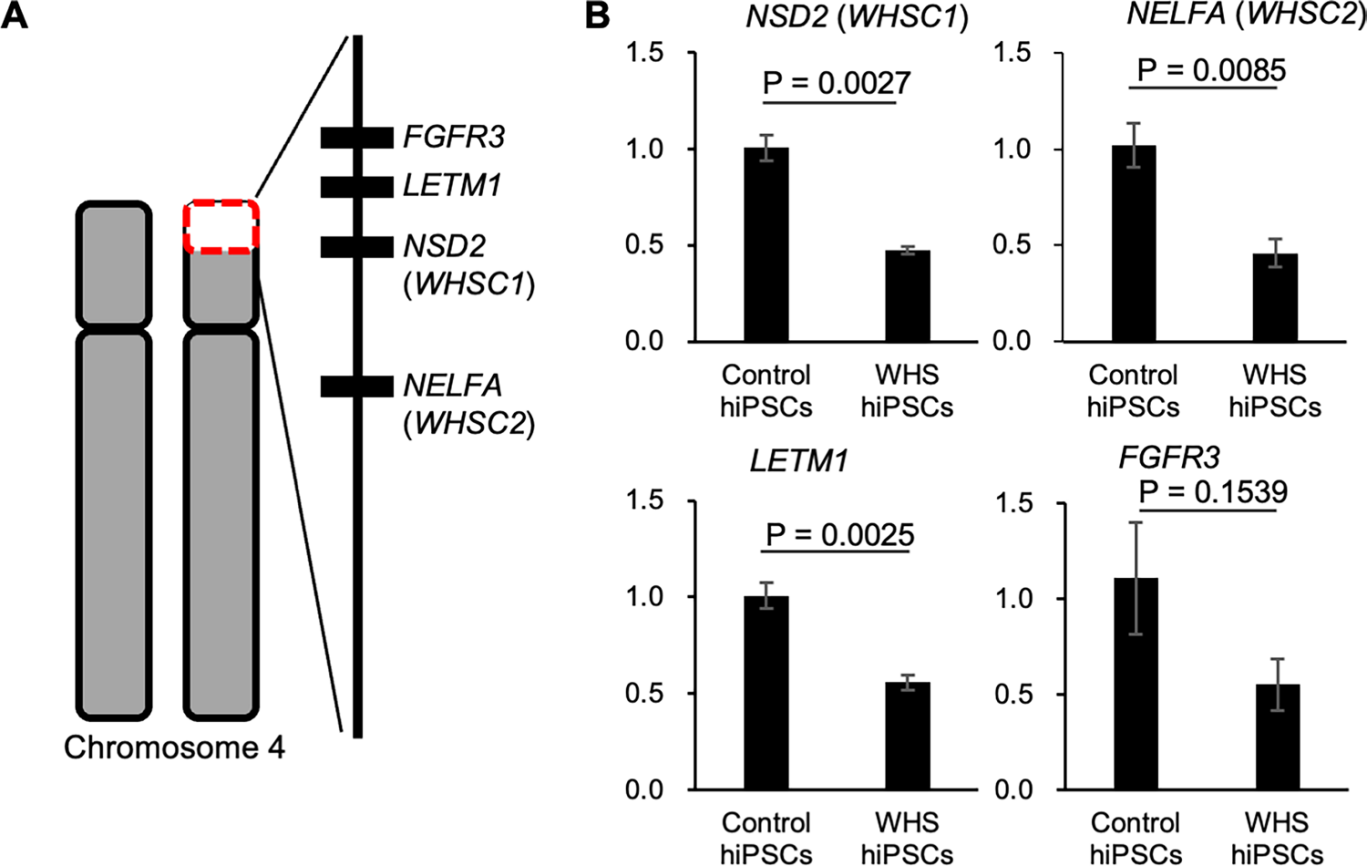
Gene expression of 4pdeleted genes in hiPSC lines derived from WHS patients. **A** Schematics of chromosomal deletion within the 4p region. Representative genes are indicated as a black bar. **B** Expression levels of representative deleted genes (*WHSC1*, *WHSC2*, *LETM1*, and *FGFR3*) detected with RT-qPCR. Data are normalized to GAPDH expression levels. P-value were calculated using Student’s *t*-test. Bar graphs show the mean ± standard errors (*n* = 4 of different hiPSC lines)

The expression levels of representative genes within the deleted 4p region were evaluated in these four WHS-specific hiPSCs with RT-qPCR, in comparison to four healthy donor-derived control hiPSC lines (WTC11, 1383D6, 19-9-7T, and 648A1). The expression levels of *WHSC1*, *WHSC2*, *LETM1*, and *FGFR3*, located in the deleted region were approximately halved in the WHS-specific hiPSCs compared to the control hiPSCs (Fig. 5B). These results indicated that hemizygous deletions in WHS-specific hiPSCs consistently affected their gene expression.

**Fig. 6:**
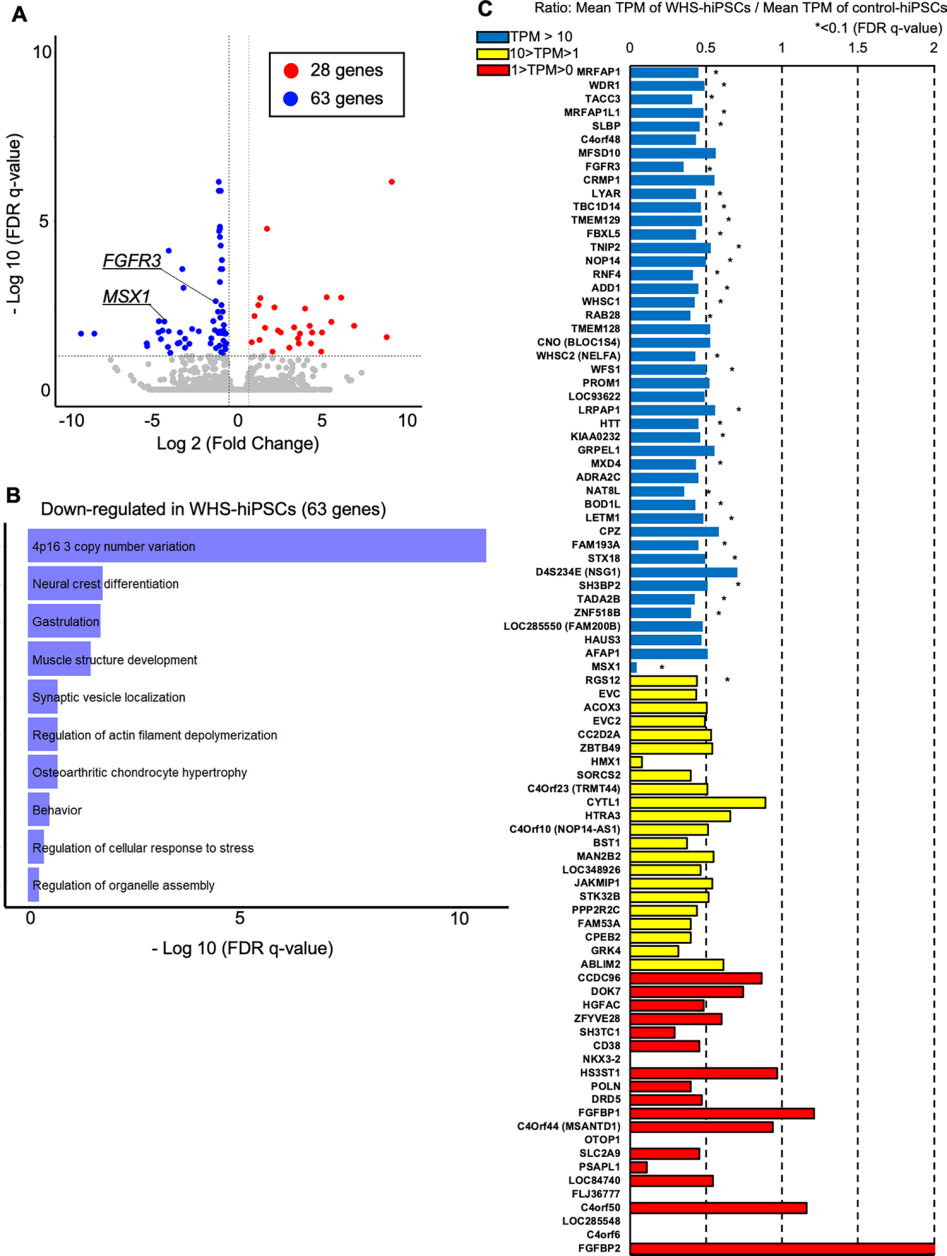
Transcriptome of hiPSC lines derived from WHS patients. **A** Volcano plot showing differential gene expression detected from RNA-seq in WHS-hiPSCs compared to control hiPSCs (*n* = 4 of different hiPSC lines). Up-regulated genes (FDR < 0.1, Fold Change > 1.5) are indicated as red dots. Down-regulated genes (FDR < 0.1, Fold Change < 1.5) are indicated as blue dots. The other genes are indicated as gray dots. **B** Pathway enrichment analysis of a gene set composed of down-regulated genes in WHS-hiPSCs. Results are ranked by − log10 (FDR *q*-value). **C** The ratio of the expression of deleted genes identified in this study. The ratio of each gene is calculated from the mean of WHS-hiPSCs compared to control hiPSCs (*n* = 4 of different hiPSC lines). Blue, yellow, and red columns show the genes of which the expression levels are 10 > TPM, 10 > TPM > 1, and 1 > TPM of the mean of control hiPSCs, respectively. Not detected genes are omitted in this bar graph. *Indicates FDR < 0.1

To examine global expression patterns in these hiPSCs, RNA-seq transcriptome analysis was performed. This analysis revealed that 28 and 63 genes were significantly up-regulated or down-regulated, respectively, in WHS-specific hiPSCs (Fig. 6A). Gene ontology and pathway analyses of these down-regulated genes unbiasedly identified “4p16.3 copy number variation” (Wikipathways, WP5365, log10(q) = − 10.48) (Fig. 6B), which is consistent with the known genetic defect of WHS. Of note, these down-regulated genes were also associated with “Neural crest differentiation” (Wikipathways, WP2064, log10(q) = − 1.71), suggesting potential defects in NCC development in these cells. We then specifically examined the gene expression levels of the 100 commonly-deleted genes in these WHS-specific hiPSCs identified by CNV array analysis (Fig. 4C). The expression of most of these genes were approximately halved in these WHS-specific hiPSCs (Fig. 6C and Supplementary Table 4). Among them, 32 genes had significantly reduced expression, particularly those with sufficient expression levels above 10 Transcripts Per Million (TPM). These significantly down-regulated genes included *WHSC1*, *WHSC2*, *LETM1*, *MSX1* and *FGFR3*, which were involved in NCC development [33–35]. These results indicate that WHS-specific hiPSCs exhibited abnormal gene expression patterns featured as 4p deletions and suggested the potential defects in NCC development.

In this study, we successfully established and characterized four WHS-specific hiPSC lines carrying 4p deletions. These hiPSC lines are valuable resources for developing disease models and the elucidating the molecular pathogenesis of WHS, particularly since the genetic and/or environmental factors to contributing to WHS symptoms are not fully understood.

## Supplementary Information

Below is the link to the electronic supplementary material.

Supplementary file1 (PDF 385 KB)

## Data Availability

The data that support the findings of this study are available on request from the corresponding author.
