# Supplementary material for: Generation of human induced pluripotent stem cell lines derived from Wolf–Hirschhorn syndrome patients with chromosomal 4p deletion
Source: Hum Cell. 2025 Sep 19;38(6):164. doi: 10.1007/s13577-025-01292-x (PMC12449351; doi:10.1007/s13577-025-01292-x)
Supplement: Supplementary file 1 — Supplementary file1 (PDF 385 KB) [file 13577_2025_1292_MOESM1_ESM.pdf]

Supplementary Information

Generation of human induced pluripotent stem cell lines derived from Wolf-Hirschhorn syndrome patients with chromosomal 4p deletion

Tomoya Shimizu[a,b], Miho Takami [a], Mami Matsuo-Takasaki[a], Michiya Noguchi[c], Yukio Nakamura[c], Tadayoshi Hayata[b], Yohei Hayashi[d]

[a] iPS Cell Advanced Characterization and Development Team, BioResource Research Center, RIKEN, 3-1-1 Koyadai, Tsukuba, Ibaraki 305-0074, Japan

[b] Department of Molecular Pharmacology, Graduate School of Pharmaceutical Sciences and Faculty of Pharmaceutical Sciences, Tokyo University of Science, 6-3-1 Niijuku, Katsushika, Tokyo, 125-8585, JAPAN

[c] Cell Engineering Division, BioResource Research Center, RIKEN, 3-1-1 Koyadai, Tsukuba, Ibaraki 305-0074, Japan.

[d] CiRA Foundation, Research and Development Center, Nakanoshima Qross 7F, 4-3-51 Nakanoshima, Kita-ku, Osaka 530-0005, Japan.

Correspondence should be sent to Yohei Hayashi (yohei.hayashi@cira-foundation.or.jp)

Table of contents

- Supplementary Figures 1 and 2
- Supplementary Tables 1 - 3
  (Supplementary Table 4 is provided as a spreadsheet)

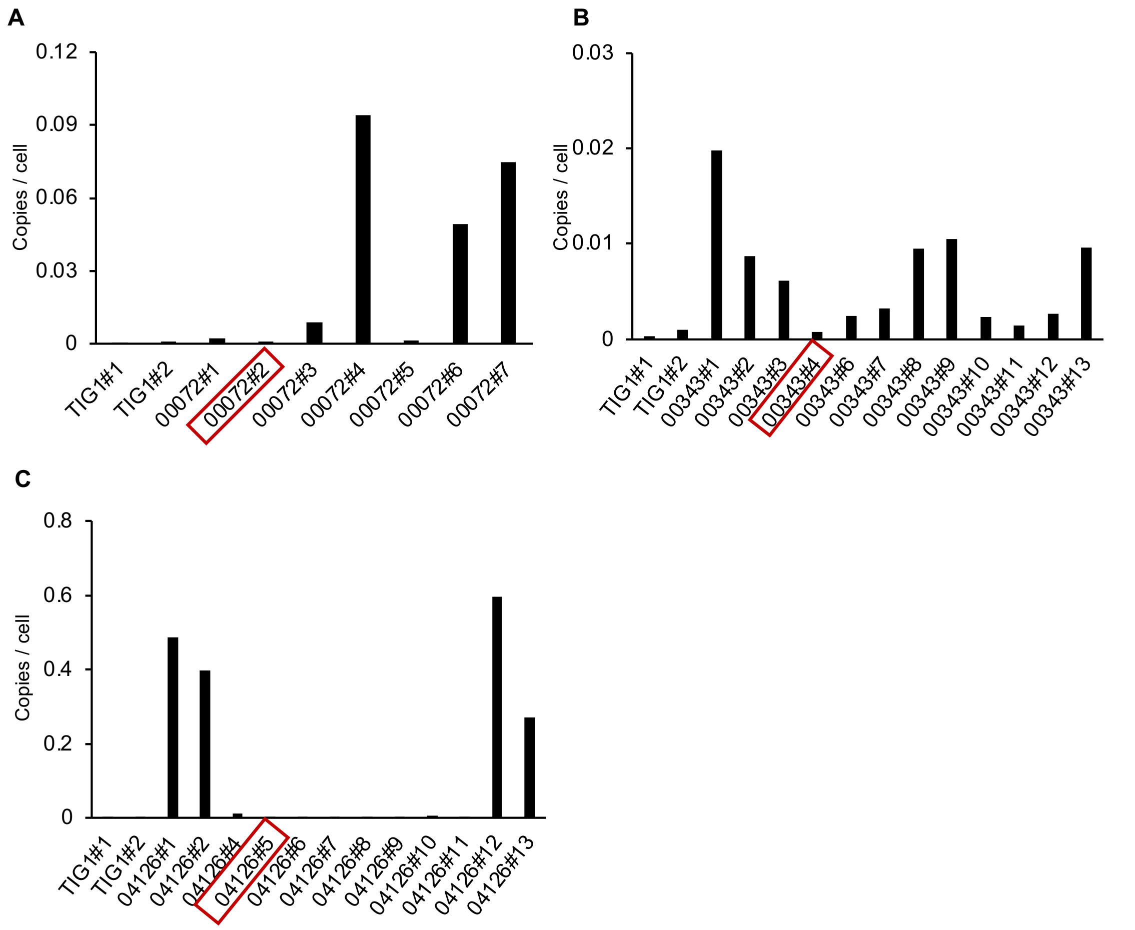

Supplementary Figure 1. Detection of episomal vector copy numbers in hiPSCs

(A-C) qPCR analysis of EBNA1 to detect residual episomal plasmid vector in HiPS-GM00072 (A), HiPS-GM00343 (B), and HiPS-GM04126 (C). # = clone number of each cell line. Red boxes indicate clones used in subsequent analyses. TIG1#1 and #2, established using SeV vector, were used as negative controls for the residual episomal plasmid vector assay.

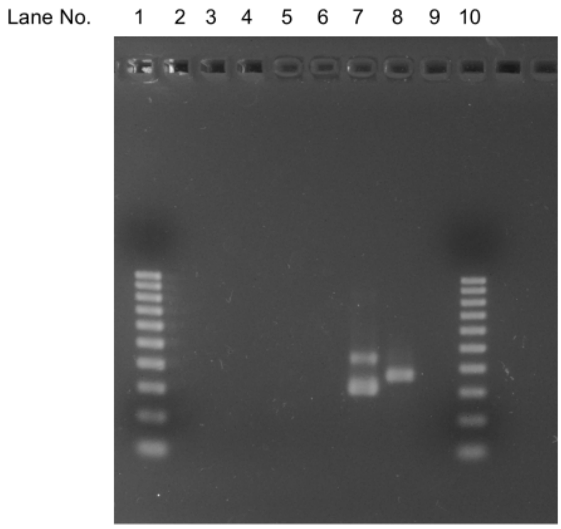

Lane No.	Sample name
1	Marker (100bp DNA Ladder)
2	Negative control
3	HiPS-GM00072
4	HiPS-GM00343
5	HiPS-GM04126
6	HPS3003
7	Positive control (Infected cell sup)
8	Positive control (DNA, Mycoplasma)
9	Negative control
10	Marker (100bp DNA Ladder)

Supplementary Figure 2. Mycoplasma contamination test

Left panel shows the electrophoresis result of nested PCR products. A summary of each lane's sample is presented in the table on the right. Nuclease free water used in the PCR mixture was loaded as negative control (Lane 2 and Lane 9). DNA extracted from the culture supernatant of mycoplasma-contaminated cells was used as positive control (Lane 7). Purified mycoplasma genomic DNA was used as positive control (Lane 8).

Supplementary Table 1: Reagent information

	**Antibodies used for immunocytochemistry and flow-cytometry**			
	**Antibody**	**Dilution**	**Company cat #**	**RRID**
Pluripotency marker	Goat anti-OCT3/4	1:200	R and D systems, Cat# AF1759	AB_354975
Pluripotency marker	Rabbit anti-NANOG	1:500	ReproCELL Incorporated, Cat# RCAB004P-F	AB_1560380
Pluripotency marker	APC anti-human SSEA-4 antibody	1:20	Biolegend, Cat# 330418	AB_2616819
Pluripotency marker	BV421 Mouse Anti-Human TRA-1-60	1:20	BD Biosciences, Cat# 562711	AB_2737738
Differentiation marker (Ectoderm)	Mouse anti-TUJ1	1:250	R and D systems, Cat# MAB1195	AB_357520
Differentiation marker (Mesoderm)	Mouse anti-SMA	1:250	R and D systems, Cat# MAB1420	AB_262054
Differentiation marker (Endoderm)	Mouse anti-AFP	1:200	R and D systems, Cat# MAB1368	AB_357658
Secondary antibody for immunocytochemistry	Donkey anti-Goat IgG Alexa Fluor 488	1:200	Thermo Fisher Scientific, Cat# A-32814	AB_2762838
Secondary antibody for immunocytochemistry	DyLight 649 Donley anti-rabbit IgG	1:500	Biolegend, Cat# 406406	AB_1575135
Secondary antibody for immunocytochemistry	Donkey anti-Mouse IgG Alexa Fluor 488	1:500	Thermo Fisher Scientific, Cat# A-21202	AB_141607

	**TaqMan probes**	
	**Target**	**TaqMan assay ID**
Housekeeping	GAPDH	Hs02786624_g1
Chromosome 4	NSD2 (WHSC1)	Hs00370212_m1
Chromosome 4	NELFA (WHSC2)	Hs00171805_m1
Chromosome 4	LETM1	Hs00360061_m1
Chromosome 4	FGFR3	Hs00179829_m1

	**Primers**			
	**Target**	**Size of band**	**Sequence (Forward primer)**	**Sequence (Reverse primer)**
Episomal vector detection	EBNA1	61	ATCAGGGCCAAGACATAGAGATG	GCCAATGCAACTTGGACGTT
Nested-PCR, 1st step PCR (MCGpF11/MCGpR1)	Mycoplasma detection	350- 850	ACACCATGGGAG(C/T)TGGTAAT	CTTC(A/T)TCGACTT(C/T)CAGACCCAAGGCAT
Nested-PCR, 2nd step PCR (R16-2/MCGpR21)	Mycoplasma detection	200- 750	GTG(C/G)GG(A/C)TGGATCACCTCCT	GCATCCACCA(A/T)A(A/T)AC(C/T)CTT

Supplementary Table 2: Summary of general clinical information for donors of WHS-specific hiPSCs

Patient number	HiPSC line name (RIKEN BRC ID)	Age at blood collection (Years old)	Sex	Ethnicity	Original donor sample	Clinical symptoms	References
1	HiPS-GM00072 (HPS5146)	11	Male	Caucasian	Fibroblast (GM00072 line)	Multiple abnormalities, including mental retardation, microcephaly, hypertelorism, oblique palpebral fissures, strabismus, and hypospadias.	[30-32]
2	HiPS-GM00343 (HPS5147)	3	Male	Unknown	Fibroblast (GM00343 line)	Multiple congenital anomalies	[31,32]
3	HiPS-GM04126 (HPS5148)	1	Male	Caucasian	Fibroblast (GM04126 line)	Unknown	[32]
4	CiRA-j-1560-c (HPS3003)	30-39	Male	Asian	PBMC	Not available in public	[20]

Supplementary Table 3: Deleted gene list in WHS-specific hiPSCs.

"x" indicates the deletion in the hiPSC line.

	HiPS-GM00072	HiPS-GM00343	HiPS-GM04126	HPS3003
ZNF595		×	×	×
ZNF718		×	×	×
ZNF876P		×	×	×
ZNF732		×	×	×
ZNF141		×	×	×
ABCA11P		×	×	×
ZNF721		×	×	×
PIGG		×	×	×
PDE6B		×	×	×
ATP5I		×	×	×
MYL5		×	×	×
MFSD7		×	×	×
PCGF3		×	×	×
CPLX1		×	×	×
GAK		×	×	×
TMEM175		×	×	×
DGKQ		×	×	×
SLC26A1		×	×	×
IDUA		×	×	×
FGFRL1		×	×	×
RNF212		×	×	×
SPON2		×	×	×
LOC100130872-SPON2		×	×	×
LOC100130872		×	×	×
CTBP1		×	×	×
C4Orf42 (CTBP1-AS2)		×	×	×
MAEA		×	×	×
KIAA1530 (UVSSA)		×	×	×
CRIPAK		×	×	×
FAM53A	×	×	×	×
SLBP	×	×	×	×
TMEM129	×	×	×	×
TACC3	×	×	×	×
FGFR3	×	×	×	×
LETM1	×	×	×	×
WHSC1	×	×	×	×
SCARNA22	×	×	×	×
WHSC2 (NELFA)	×	×	×	×
MIR943	×	×	×	×
C4Orf48	×	×	×	×
NAT8L	×	×	×	×
POLN	×	×	×	×
HAUS3	×	×	×	×
MXD4	×	×	×	×
ZFYVE28	×	×	×	×
RNF4	×	×	×	×
FAM193A	×	×	×	×
TNIP2	×	×	×	×

SH3BP2	×	×	×	×
ADD1	×	×	×	×
MFSD10	×	×	×	×
C4Orf10 (NOP14-AS1)	×	×	×	×
NOP14	×	×	×	×
GRK4	×	×	×	×
HTT	×	×	×	×
C4Orf44 (MSANTD1)	×	×	×	×
RGS12	×	×	×	×
HGFAC	×	×	×	×
DOK7	×	×	×	×
LRPAP1	×	×	×	×
ADRA2C	×	×	×	×
LOC348926	×	×	×	×
OTOP1	×	×	×	×
TMEM128	×	×	×	×
LYAR	×	×	×	×
ZBTB49	×	×	×	×
D4S234E (NSG1)	×	×	×	×
STX18	×	×	×	×
MSX1	×	×	×	×
CYTL1	×	×	×	×
STK32B	×	×	×	×
C4Orf6	×	×	×	×
EVC2	×	×	×	×
EVC	×	×	×	×
CRMP1	×	×	×	×
C4Orf50	×	×	×	×
JAKMIP1	×	×	×	×
WFS1	×	×	×	×
PPP2R2C	×	×	×	×
MAN2B2	×	×	×	×
MRFAP1	×	×	×	×
LOC93622	×	×	×	×
S100P	×	×	×	×
MRFAP1L1	×	×	×	×
CNO (BLOC1S4)	×	×	×	×
KIAA0232	×	×	×	×
TBC1D14	×	×	×	×
CCDC96	×	×	×	×
TADA2B	×	×	×	×
GRPEL1	×	×	×	×
FLJ36777	×	×	×	×
SORCS2	×	×	×	×
PSAPL1	×	×	×	×
LOC84740	×	×	×	×
AFAP1	×	×	×	×
ABLIM2	×	×	×	×
SH3TC1	×	×	×	×
HTRA3	×	×	×	×
ACOX3	×	×	×	×
C4Orf23 (TRMT44)	×	×	×	×

CPZ	×	×	×	×
HMX1	×	×	×	×
LOC650293	×	×	×	×
USP17	×	×	×	×
USP17L6P	×	×	×	×
DEFB131	×	×	×	×
MIR548I2	×	×	×	×
DRD5	×	×	×	×
SLC2A9	×	×	×	×
WDR1	×	×	×	×
ZNF518B	×	×	×	×
CLNK	×	×	×	×
MIR572	×	×	×	×
HS3ST1	×	×	×	×
HSP90AB2P	×	×	×	×
RAB28	×	×	×	×
NKX3-2	×	×	×	×
LOC285548	×	×	×	×
BOD1L	×	×	×	×
CPEB2	×	×	×	×
C1QTNF7	×	×	×	×
CC2D2A	×	×	×	×
FBXL5	×	×	×	×
LOC285550 (FAM200B)	×	×	×	×
BST1	×	×	×	×
CD38	×	×	×	×
FGFBP1	×	×	×	×
FGFBP2	×	×	×	×
PROM1	×	×	×	×
TAPT1	×	×	×	
FLJ39653 (TAPT1-AS1)	×	×	×	
LDB2	×	×	×	
QDPR	×	×	×	
CLRN2	×	×	×	
LAP3	×	×	×	
MED28	×	×	×	
FAM184B	×	×	×	
DCAF16	×	×	×	
NCAPG	×	×	×	
LCORL	×	×	×	
SLIT2	×	×	×	
MIR218-1	×	×	×	
PACRGL	×	×	×	
KCNIP4	×	×	×	
NCRNA00099	×	×	×	
GPR125	×	×	×	
GBA3	×	×	×	
PPARGC1A	×	×	×	
MIR573	×	×	×	
DHX15	×	×	×	
SOD3	×	×	×	
CCDC149	×	×	×	

LGI2	×	×	×	
SEPSECS	×	×	×	
PI4K2B	×	×	×	
ZCCHC4	×	×	×	
ANAPC4	×	×	×	
SLC34A2	×	×	×	
SEL1L3	×	×	×	
C4Orf52 (SMIM20)		×	×	
RBPJ		×	×	
CCKAR		×	×	
TBC1D19		×	×	
STIM2		×	×	
